# Meta-Analysis of the Effect of Saline-Alkali Land Improvement and Utilization on Soil Organic Carbon

**DOI:** 10.3390/life12111870

**Published:** 2022-11-13

**Authors:** Shuai Yang, Xinghai Hao, Yiming Xu, Juejie Yang, Derong Su

**Affiliations:** School of Grassland Science, Beijing Forestry University, Beijing 100083, China

**Keywords:** soil saline-alkalization, improvement and utilization, national scale, meta-analysis, carbon stocks

## Abstract

There is a large amount of saline-alkali land in China. Through the improvement and utilization of saline-alkali land to improve the carbon content in soil, it can not only become a reserve resource of cultivated land or grazing grassland, but also become an important land “carbon sink”. In this study, we performed a comprehensive meta-analysis to identify the impact of improvement and utilization of saline-alkali soil on soil organic carbon (SOC) in China. Our results showed that the soil salt and alkali content in Heilongjiang Province and Jilin Province in China was the highest, with an SOC content between 3.05 and 17.8 g/kg and pH between 8.84 and 9.94. Among the five methods of reclamation, halophyte planting, fertilization, biochar and modifier application, only biochar and modifier application significantly increased the SOC content (*p* < 0.05). The content of SOC in saline-alkali soil was 2.9–6.3 g/kg before biochar application, and significantly increased to 6.2–13.05 g/kg after biochar application (*p* < 0.01). The SOC content was 3.05–8.12 g/kg before the application of the modifier, and significantly increased to 3.68–9 g/kg (*p* < 0.05) after the application of the modifier. After utilization and improvement of saline-alkali land, the total nitrogen, available phosphorus and available potassium also increased significantly (*p* < 0.05). This study provides a scientific basis for further understanding the improvement and utilization of saline-alkali land in China and its potential for increasing carbon sinks.

## 1. Introduction

Saline-alkali land is widely distributed on the earth, and the area of saline-alkali land in the world is about 1.0 × 10^9^ hm^2^, accounting for about 25% of the earth’s land area and 76% of the world’s cultivated land area [1]. The saline-alkali land area in China is about 9.9 × 10^7^ hm^2^, mainly distributed in the north, including Xinjiang, Gansu, Qinghai, Inner Mongolia, Ningxia Province and coastal areas [1]. As a special land resource, saline-alkali land has a great potential to be utilized. Large areas of saline-alkali land in China have been abandoned for a long time. How to improve the utilization efficiency of saline-alkali land so as to achieve carbon sequestration and sink increase is an urgent problem that needs to be solved [2].

The saline-alkali land in North China Plain is mainly cultivated for commercial crops by enhancing the conditions of farmland water conservancy projects. In the west of Northeast China and coastal saline-alkali land, ecological grass is mainly constructed, and halophytes are planted to develop animal husbandry [3]. The physical improvement measures of saline-alkali land mainly include the rectification of the original soil layer, enhancement of exotic soil, micro area soil improvement and ground cover, as well as irrigation and salt washing, drainage and desalination, water saving and salt control, etc. [4,5]. Chemical improvement measures are mainly to apply chemical modifiers, including gypsum, desulfurized gypsum, pyrite, and water-retaining agent, etc. [6]. While chemical improvement can effectively reduce the soil salt content, there are negative impacts such as single effect, short duration, and secondary pollution [7]. Many studies have focused on the fertilization and application of biochar to improve saline-alkali soil [8,9,10]. Green manure is a pollution-free organic fertilizer made from organic wastes such as corn straw or animal manure [4]. Increasing the application of green manure can increase soil organic matter content, improve soil structure and rhizosphere microenvironment, and facilitate the activities of soil microorganisms, thus improving soil fertility and inhibiting salt accumulation [4].

Soil organic carbon is closely related to soil fertility, and SOC pool is also an important terrestrial carbon pool. Saline-alkali soil will absorb and retain a large amount of carbon in the process of utilization and improvement, and large areas of saline-alkali land in China will have huge carbon sink potential [8]. The scientific improvement and utilization of saline-alkali land can significantly improve the function of saline-alkali land in increasing the sink of organic carbon and play an important role in achieving the goal of carbon peaking and carbon neutralization in China. However, the research on the impact of saline-alkali land improvement and utilization on SOC is relatively few in China. It is urgent to carry out comprehensive analysis on different improvement and utilization methods and changes in SOC content on a large scale.

Based on meta-analysis, response ratio analysis and univariate analysis of variance were used to quantitatively characterize the impact of utilization (reclamation and halophyte planting) and improvement (fertilization, biochar application and modifier application) on SOC content in saline-alkali land. Reclamation refers to a series of tillage measures to treat the saline-alkali land, including different farming methods and different farming times. Halophyte refers to the measures of planting saline-alkali tolerant plants in the saline-alkali land to control the saline-alkali land. Fertilization is the method of applying different fertilizers to treat saline-alkali land. Biochar is the method of treating saline land with different biochar. Modifier refers to the method of applying different modifiers to the saline land to control the saline land. This study provides a scientific basis for further understanding the improvement and utilization of saline-alkali land and its potential for increasing carbon sinks in China.

## 2. Materials and Methods

### 2.1. Data Sources

We searched journal articles published before 2021 using the Web of Science and China National Knowledge Infrastructure database, using the following keywords: saline-alkali land, soil organic carbon, salinization and soil organic carbon. To avoid bias, the following three criteria were used to choose the papers: (1) the research was based on the experiment of improving and utilizing saline-alkali land; (2) the literature contains the relevant data of soil saline-alkali content, soil organic carbon content and various soil physical and chemical properties before and after the improvement and utilization of saline-alkali land; (3) the research area is in China and the mean, standard error and sample size (n) of variables could be extracted from the chosen study. A total of 35 valid articles met the selection criteria, the final dataset included 61 sets of data (Table 1). Of the 35 articles, 6 were reclamation, 6 were halophyte, 6 were fertilization, 8 were biochar, and 9 were modifier. In our investigation, the trial period of saline-alkali land improvement and utilization was mainly 1~3 years, and only a few cases were excluded from this scope.

### 2.2. Data Extraction

The indexes extracted in this study include saline-alkali land improvement and utilization mode, soil salt content, soil organic carbon content, study area distribution and various soil physical and chemical properties. “Soil organic carbon content” is the average SOC content of 0–20 cm surface layer. The data is directly obtained from the tables and text of the literature, and the picture data is extracted by Get Data software. If the data in the literature are organic matter content, it is converted into soil organic carbon content data according to organic matter (g/kg) = soil organic carbon (g/kg) × 1.724 (van Bemmelen factor) [11]. We also extracted other soil physical and chemical properties from the literatures for analysis, such as pH, electrical conductivity (EC), exchangeable sodium percentage (ESP), water content, soil bulk density, total nitrogen (TN), available phosphorus, available potassium, and available nitrogen.

### 2.3. Statistical Analysis

In this study, response ratio analysis was used to compare the difference of SOC content before and after improvement and utilization of saline-alkali land. The formula was as follows:

*RR*= ln*X_t_* − ln*X_e_*, (1)

where *X_t_* and *X_e_* are the mean values before and after improved utilization, respectively.

If *RR* was equal to 0, it meant that the improved utilization had not caused the change of SOC content; if *RR* was less than 0, it meant that improving utilization reduced SOC content, which showed negative effect; if *RR* was greater than 0, it reflected the improvement and utilization to increase SOC content, showing a positive effect. Here, Rosenthal’s fail-safe number was 128,619 (larger than 5 k + 10 = 315), which indicated that there was no publication bias in our study [12]. SPSS 25.0 statistical software was used for data analyses, and Origin 2021 software was used for analysis and drawing.

## 3. Results

### 3.1. Regional Distribution of Soil Organic Carbon in Saline-Alkali Soil in China

**Figure 1:**
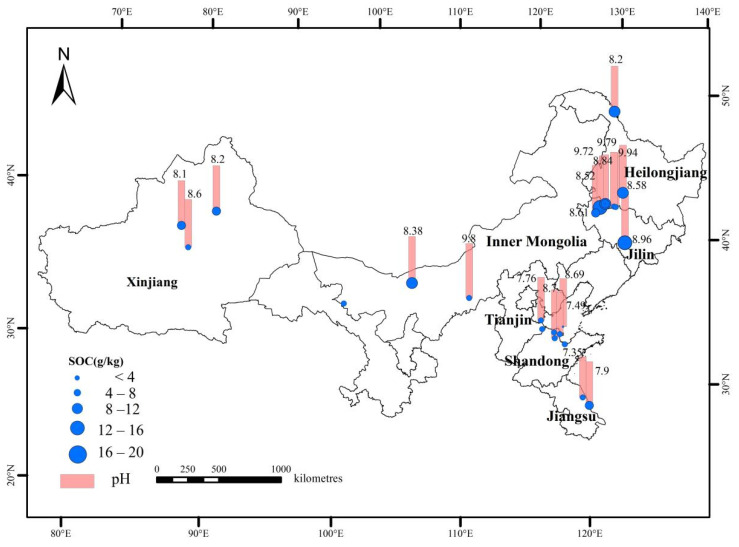
Distribution of soil organic carbon in saline-alkali soil in China.

At present, domestic research on soil organic carbon in saline-alkali land are mainly concentrated in Xinjiang, Inner Mongolia, Heilongjiang, Jilin, Tianjin, Shandong and Jiangsu Province. The vegetation types included alpine meadow, plain desert grassland, mountain meadow grassland and mountain grassland. The soil types included chestnut soil, grey desert soil, black soil, chernozem soil and soda saline-alkali soil (Figure 1). Heilongjiang Province and Jilin Province had the highest soil salt and alkali content, with a pH range of 8.84–9.94, and the highest SOC content (3.05–17.8 g/kg).

### 3.2. Response Characteristics of SOC to Improvement and Utilization

**Figure 2:**
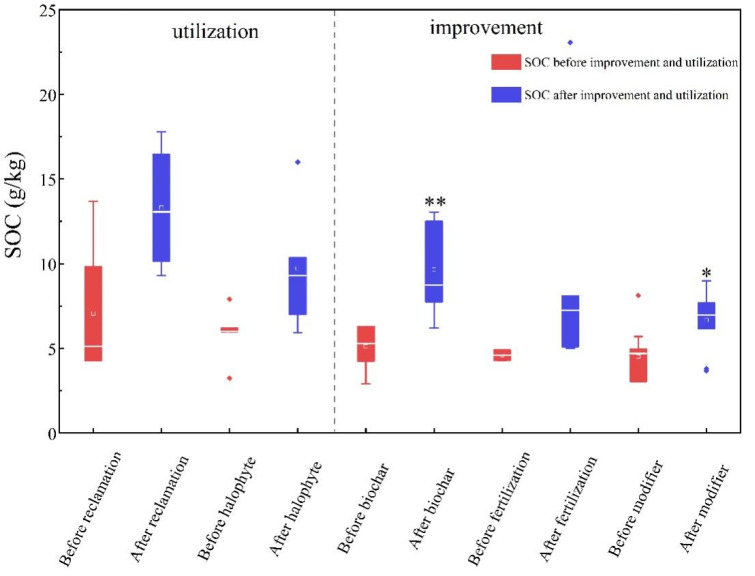
Changes of SOC content in saline-alkali soil after utilization and improvement. Significance: ** *p* < 0.01, * *p* < 0.05.

**Figure 3:**
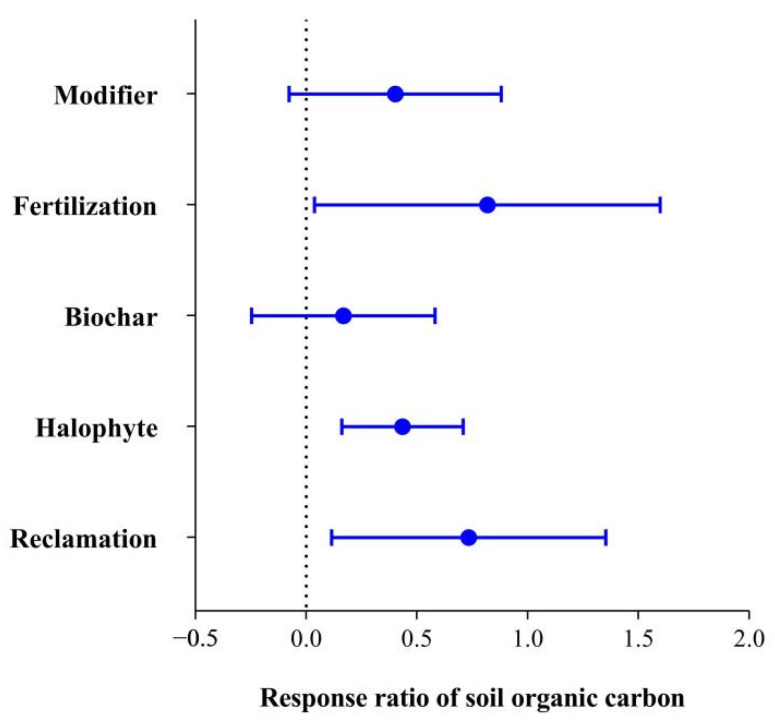
Response of SOC to improved utilization.

The SOC of saline-alkali land after reclamation, planting halophytes and fertilization increased, but it did not change significantly compared with that before improvement and utilization (*p* > 0.05). SOC in saline-alkali soil increased significantly after biochar and modifier application (Figure 2). The content of SOC in saline-alkali soil was 2.9–6.3 g/kg before biochar application, and it significantly increased to 6.2–13.05 g/kg after biochar application (*p* < 0.01). The SOC content was 3.05–8.12 g/kg before the application of the modifier, and significantly increased to 3.68–9 g/kg (*p* < 0.05) after the application of the modifier (Figure 2). The 95% confidence interval of the response ratio (*RR*) of SOC ranged from 0.48–0.98, 0.5–1.13, 0.32–0.55, 0.21–059 and 0.001–0.33 for reclamation, fertilization, halophyte planting, modifier and biochar, respectively, indicating there was a positively significant (*p* < 0.05) impact on SOC (Figure 3).

### 3.3. The Relationship between SOC and Other Physicochemical Properties in Saline-Alkali Soil

**Figure 4:**
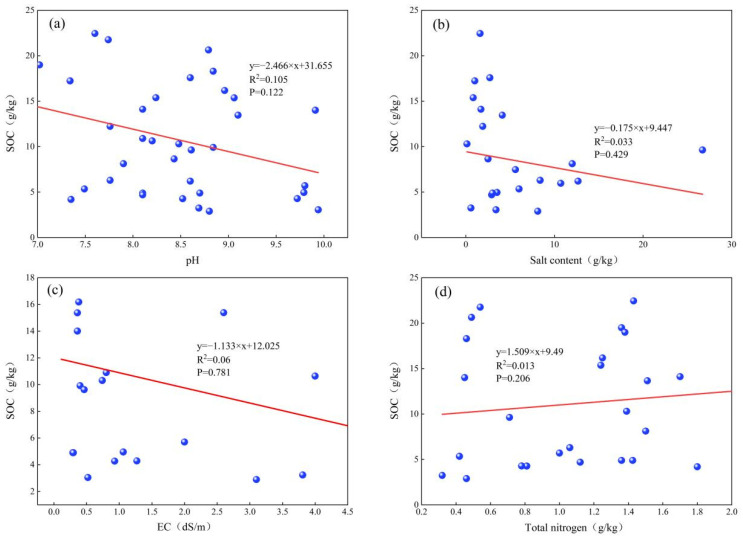
The relationships of SOC and pH, salt content, EC and TN.

On a large scale, SOC in saline-alkali land was negatively correlated with pH, salt content and electrical conductivity (EC), and positively correlated with total nitrogen (TN), but the correlation was not significant (*p* > 0.05, Figure 4).

Pearson correlation analysis showed that SOC was negatively correlated with salt content, pH, soil bulk density, exchangeable sodium percentage, electrical conductivity and available potassium, but the results are not significant (*p* > 0.05). There was a significantly positive correlation between SOC and available nitrogen (*p* < 0.05, Table 2). After utilization and improvement, the pH, electrical conductivity and soil bulk density decreased, while the total nitrogen, available phosphorus and available potassium increased (Table 3).

## 4. Discussion

### 4.1. The Relationship between the Type and Content of Saline Alkali and SOC

At present, domestic research on soil organic carbon in saline-alkali land are mainly conducted in coastal areas such as Xinjiang, Inner Mongolia, Heilongjiang, Jilin, Tianjin, Shandong and Jiangsu Province. Northeast China is an inland soda saline-alkali land. The soil is mainly carbonate and bicarbonate, pH > 8.5, alkaline. North China is a secondary saline-alkali land in Hetao irrigation area, with chloride and sulfate as the main salt, and some cracked alkaline land. Northwest China is an inland saline-alkali land, and the salt is mainly chloride and sulfate. Saline-alkali land in North China is dominated by chloride and sulfate, and saline-alkali land with different salt types is distributed alternately [13]. The coastal area is saline-alkali land of coastal beach, and the salt is mainly sodium chloride [13].

Hydrogeological and hydrochemical conditions are one of the important causes of soil salinization in China. Ying Zhao et al. found that coastal areas were mainly affected by sea tide and groundwater [14]. Most coastal areas have flat terrain, stagnant surface water, slow groundwater runoff and shallow groundwater level. In addition, the ineffective evaporation of surface water is strong, and the surface salinization of soil is heavy, which often leads to soil hardening and crusting. As a result, the soil has high salt content, high pH and low organic matter content. Soil salinization destroys soil structure, reduces SOC sequestration capacity, and thus leads to the decrease of SOC [14]. Xu Xiaohong’s research showed that hydrogeology and hydrochemical conditions played an important driving role in the occurrence of soil salinization in soda saline-alkali land in Northeast China, and the main influencing factors are groundwater salinity, groundwater depth, surface and underground runoff intensity. The groundwater in Songnen Plain is shallow (about 1.5~3 m), and its salinity is high (usually 2~5 g/L, up to 10 g/L), which is conducive to the surface aggregation of soil salt [15]. The accumulation of salt in soil destroyed the soil structure and soil aggregates. The research of Zhang Hao et al. shows that increasing the amount of soil aggregates is helpful to increase the content of soil organic carbon [8]. In contrast, the number of soil aggregates decreased during the formation of soda saline-alkali land in Northeast China, resulting in the decrease of SOC.

### 4.2. Effect of Saline-Alkali Land Improvement on SOC

Meta-analysis showed that compared with reclamation, planting halophytes and fertilization, the SOC of saline-alkali land improved by biochar and modifier increased significantly. This is mainly due to the fact that biochar is a porous material rich in carbon, which is produced by biomass pyrolysis at a relatively low temperature (<700 °C) under anaerobic or limited oxygen conditions. It has some special characteristics, including porous structure, high specific surface area and special physical and chemical properties [10]. It can obviously improve the bulk density and pore structure of soil and promote the formation of aggregates. At the same time, biochar adsorbs and fixes mineral nutrients in soil, which helps to improve soil and promote carbon accumulation [13]. The research results obtained by Kang et al. in the experiment of improving coastal saline-alkali land with biochar showed that the application of biochar significantly improved the physical and chemical properties of saline-alkali soil and reduced the damage of saline-alkali stress to the growth of Miscanthus plants. Especially in the experiment, compared with the control group, the content of soil organic matter in the experimental group increased by 4.5–4.9 times after adding biochar, which greatly enhanced the carbon fixation capacity of the soil [10]. In addition, biochar itself is a kind of carbon, accounting for about 70~80% of the total carbon, and its structure is relatively stable aromatic carbon and aliphatic carbon. The research of Liu Miao et al. shows that after biochar improves saline-alkali land, SOC content significantly increases. Moreover, the abundant microporous structure and large specific surface area of biochar are beneficial to the survival and reproduction of microorganisms, increase the number of beneficial bacteria in soil, enhance the function of soil ecosystem, and provide a good growth environment for crop roots [13]. Chen Wenfu et al.’ s research shows that high carbon content, rich pore structure, large specific surface area and stable physical and chemical properties are the inherent characteristics of biochar, and they are also the important structural basis for biochar to be returned to fields to improve soil, increase crop yield and realize carbon sequestration [16].

The modifiers used in this statistical literature are organic modifiers, mainly organic wastes such as straw, manure, perishable garbage, etc. These organic modifiers have higher contents of C, N, P, K and other nutrients, which are similar to biochar. Therefore, the improvement results of organic modifiers on saline-alkali land are very similar to those of biochar.

Modifier and biochar increased SOC by promoting growth of halophytes and increasing soil enzyme activity. Halophytes and microorganisms had a mutual relationship to resist saline-alkali stress through plant rhizosphere interaction, because plants could provide food for microorganisms and microorganisms could mobilize mineral bound nutrients for plants through chelation, complexation and dissolution processes in saline-alkali soil [17]. Plants secreted low-molecular weight organic compounds into the rhizosphere to shape the surrounding microbial communities, which could enrich different microbial groups and promote their activities [18]. Cell wall polymers from microorganisms were potentially important sources of long-term stability of soil carbon [19]. Modifier and biochar stimulating microbial activity and enzyme activity could increase the accumulation of soil carbon [20].

### 4.3. Changes of Physical and Chemical Properties of Soil after Saline-Alkali Land Improvement

This study shows that after the improvement and utilization of saline-alkali land, the pH, electrical conductivity and soil bulk density of soil physical and chemical properties all decrease, while the total nitrogen, available phosphorus and available potassium significantly increase. Liang Jiaping’s research shows that planting halophytes to improve saline-alkali land reduces soil salt content, bulk density, Na^+^ content and pH value, and increases soil porosity, soil organic carbon and N, P, K and other nutrients [21]. The growth of halophytes needs osmotic adjustment by absorbing salt ions in saline soil solution, thus reducing the soil salt content and pH value [22]. Halophytes can also improve the physical and chemical properties of soil affected by salt, absorb more N, P, K and other nutrients through root growth, and fix them in the soil [23]. In the aspect of biological improvement of saline-alkali soil, the research of Liu Miao et al. shows that due to the low bulk density, high porosity and strong water holding capacity of biochar, the physical dilution effect can reduce soil bulk density and improve soil pore condition, thus improving soil water permeability, speeding up salt leaching and reducing soil salt content and pH value; The Ca^2+^ and Mg^2+^ ions contained in carbon can exchange Na^+^ adsorbed by saline-alkali soil colloids, thus preventing colloid dispersion, promoting soil particle cementation and flocculation, and promoting the formation of soil aggregate structure and improving soil structure through electrostatic bonding of multivalent cations. Kong Xiangqing’s research also found that biochar can improve saline-alkali soil and increase the contents of available phosphorus, available potassium, total nitrogen, total phosphorus, total potassium and organic matter in saline-alkali soil.

## 5. Conclusions

Based on meta-analysis, the research on soil organic carbon in saline-alkali land in China is mainly conducted in Xinjiang, Inner Mongolia, Heilongjiang, Jilin, Tianjin, Shandong and Jiangsu, among which the salinization degree in Heilongjiang and Jilin is the most serious and concentrated, and their soil types are black soil and chernozem, with pH ranging from 8.85 to 9.94 and SOC content ranging from 3.05 to 22.45 g/kg. On the national scale, the SOC content of saline-alkali land decreases with the increase of pH, salt content and electrical conductivity, and increases with the increase of total nitrogen. The results of five typical ways of saline-alkali soil improvement and utilization, such as reclamation, planting halophytes, fertilization and application of biochar and improver, showed that the application of biochar and modifier significantly increased the SOC content (*p* < 0.05). After utilization and improvement of saline-alkali land, the total nitrogen, available phosphorus and available potassium also increased significantly (*p* < 0.05). This study provides a scientific basis for further understanding the improvement and utilization of saline-alkali land and its carbon sink potential in China.

## Figures and Tables

**Table 1:** List of collected data about effects of saline-alkali land improvement and utilization on soil organic carbon in China.

Study Area	Latitude Longitude	Land Use Type	Soil Type	pH	Average Organic Carbon Content (g/kg)
Shanghai	121°54′ E, 31°34′ N	Degraded grassland	Chestnut soil	8.1	4.96
Xinjiang	87°56′ E, 44°17′ N	Desert	Chestnut soil	8.2	10.64
Xinjiang	87°56′ E, 44°17′ N′	Desert	Chestnut soil	8.2	9.16
Jilin	124°22′ E, 45°46′ N	Farmland	Soda saline-alkali soil	9.2	13.67
Jilin	123°221′ E, 44°461′ N	Farmland	Chestnut soil	9.1	15.5
Xinjiang	85°08′ E, 42°51′ N	Farmland	Grey desert soil	8.1	10.09
Jilin	125°18′ E, 45°28′ N	Farmland	Chestnut soil	9.72	4.29
Jilin	124°68′ E, 44°91′ N	Degraded grassland	Soda saline-alkali soil	9.79	4.96
Gansu	100°30′ E, 39°42′ N	Farmland	Grey desert soil	8.9	6.01
Inner Mongolia	106°20′ E, 41°18′ N	Farmland	Chestnut soil	8.2	13.27
Jilin	124°1′ E, 45°19′ N	bare land	Soda saline-alkali soil	9.94	3.05
Jiangsu	120°22′ E, 33°32′ N	Farmland	Chestnut soil	8.5	12
Jilin	126°11′ E, 46°18′ N	Farmland	Chestnut soil	9.41	14.74
Jilin	124°22′ E, 45°46′ N	Farmland	Chestnut soil	9.45	9.92
Jilin	124°1′ E, 45°19′ N	Farmland	Soda saline-alkali soil	9.94	1.68
Shandong	118°31′ E, 37°54′ N	Farmland	Chestnut soil	8.3	6
Xinjiang	86°12′ E, 41°36′ N	Farmland	Chestnut soil	8.5	6.2
Shandong	119°20′ E, 37°04′ N	Coastal wetland	Chestnut soil	8.9	6
Shandong	118°59′ E, 37°45′ N	Coastal wetland	Chestnut soil	7.49	5.35
Shandong	37° 03′ E, 119°39′ N	bare land	Chestnut soil	8.8	2.9
Jilin	123°51′ E, 45°35′ N	Farmland	Chestnut soil	9.63	17.8
Jilin	124°1′ E, 45◦19′ N	Farmland	Chestnut soil	9.94	1.68
Hebei	117°33′ E, 38°9′ N	Coastal wetland	Chestnut soil	9.1	10.36
Jilin	123°21′ E, 45°16′ N	Wetland	Chestnut soil	8.63	9.63
Jilin	125°18′ E, 45°28′ N	Farmland	Chestnut soil	9.3	7.91
Jilin	124°1′ E, 45°19′ N	Farmland	Chestnut soil	8.9	1.68
Jilin	124°03′ E, 45°05′ N	Wetland	Chestnut soil	7.52	16.28
Shandong	119°20′ E, 38°12′ N	Farmland	Chestnut soil	8.62	3.24
Jiangsu	120° 49′ E, 32°59′ N	Coastal wetland	Chestnut soil	7.9	8.12
Tianjin	117°30′ E, 38°44′ N	Wetland	Chestnut soil	7.76	3.65
Shandong	118°32′ E, 37°31′ N	Farmland	Chestnut soil	8.4	4.9
Inner Mongolia	111°23′ E, 40°23′ N	Farmland	Chestnut soil	9.8	5.7
Shandong	118°32′ E, 37°31′ N	Farmland	Chestnut soil	8.1	4.7
Jilin	125°38′ E, 43°05′ N	Farmland	Chestnut soil	8.96	16.18
Shandong	118°32′ E, 37°31′ N	Farmland	Chestnut soil	8.89	4.9

**Table 2:** Pearson correlation analysis table between SOC and various factors in soil.

	SOC (g/kg)
Salt content (g/kg)	−0.182
PH	−0.299
Soil bulk density (g/cm^3^)	−0.391
ESP (%)	−0.182
EC (dS/m)	−0.078
Organic matter (g/kg)	0.570
water content (%)	0.132
Total nitrogen (g/kg)	0.313
Available nitrogen (mg/kg)	0.787 *
Total phosphorus (g/kg)	0.330
Available phosphorus (mg/kg)	0.103
Available potassium (mg/kg)	−0.326

Significance: * *p* < 0.05.

**Table 3:** Changes of soil physical and chemical properties after saline-alkali land utilization and improvement.

Physical and Chemical Properties of Soil	Before Utilization	After Utilization
pH	9.32 ± 0.54	8.26 ± 0.57
Electrical conductivity (dS/m)	5.10 ± 4.84	0.36 ± 0.22
Exchangeable sodium percentage (%)	27.04 ± 0.01	2.18 ± 3.06
Water content (%)	41.40 ± 0.01	52.40 ± 5.51
Soil bulk density (g/cm^3^)	1.37 ± 0.01	1.21 ± 0.02
Total nitrogen (g/kg)	0.32 ± 0.12	0.84 ± 0.32
Available phosphorus (mg/kg)	5.66 ± 1.53	12.28 ± 11.11
Available potassium (mg/kg)	172.0 ± 0.01	231.0 ± 108.89
**Physical and chemical properties of soil**	**Before improvement**	**After improvement**
pH	8.58 ± 0.50	8.10 ± 0.88
Electrical conductivity (dS/m)	0.32 ± 0.01	0.29 ± 0.05
Soil bulk density (g/cm^3^)	1.49 ± 0.16	1.45 ± 0.16
Total nitrogen (g/kg)	0.85 ± 0.08 **	1.51 ± 0.23
Available nitrogen (mg/kg)	28.81 ± 0.27 **	49.91 ± 9.34
Total phosphorus (g/kg)	0.19 ± 0.01 **	0.75 ± 0.25
Available phosphorus (mg/kg)	8.75 ± 0.27 *	18.15 ± 8.89
Available potassium (mg/kg)	163.68 ± 30.96 **	243.13 ± 51.27

Significance: ** *p* < 0.01, * *p* < 0.05.

## Data Availability

Not applicable.
